# Understanding the heterogeneity of cervical cancer screening non-participants: Data from a national sample of British women

**DOI:** 10.1016/j.ejca.2017.04.017

**Published:** 2017-07

**Authors:** Laura A.V. Marlow, Amanda J. Chorley, Jessica Haddrell, Rebecca Ferrer, Jo Waller

**Affiliations:** a Cancer Communication & Screening Group, Research Department of Behavioural Science and Health, UCL, Gower Street, London, WC1E 6BT, UK; b Basic Biobehavioral and Psychological Sciences Branch, Behavioral Research Program, Division of Cancer Control and Population Sciences, National Cancer Institute, Rockville, MD, USA

**Keywords:** Cervical cancer screening, Intention, Uptake, Interventions, PAPM, Inequalities, Age, Stages, Readiness

## Abstract

**Background:**

Uptake of cervical cancer screening in the United Kingdom (UK) is falling year on year, and a more sophisticated understanding of non-participation may help design interventions to reverse this trend. This study ascertained the prevalence of different non-participant types using the Precaution Adoption Process Model (PAPM).

**Methods:**

Home-based computer-assisted interviews were carried out with 3113 screening-eligible women in Britain. Survey items assessed self-reported screening uptake and intention to attend in future. Responses to these items were used to classify women into one of five different types of non-participants.

**Results:**

Of 793 non-participants, 28% were unaware of screening, 15% had decided not to attend and 51% were intending to have screening but were currently overdue. Younger women were more likely to be unaware of screening or to intend to be screened, while older women were more likely to have decided not to be screened. Women from ethnic minority backgrounds were more likely to be unaware of screening than white women. Being in a lower social grade was associated with increased odds of all three types of non-participation.

**Conclusion:**

The majority of cervical cancer screening non-participants are not making an active decision not to attend but rather are either unaware or unable to act. There are clear sociodemographic differences between non-participant types, which could be used to identify where tailored interventions may be best targeted.

## Highlights

• We have identified three dominant types of cervical cancer screening non-participants.
• Over half of those who are not up to date with screening intend to be screened.
• Women from ethnic minority backgrounds are more likely to be unaware of screening.
• Most non-participants are not making an active decision not to attend.
• Targeting interventions at non-participant groups could improve their success.

## 1 Introduction

Cancer screening offers the opportunity to detect asymptomatic cancer or precancer (e.g. dysplasia or polyps) in those who appear and feel healthy. This can improve treatment outcomes and reduce morbidity and mortality [1]. Many European countries have organised screening programmes, which use population-based registers to ensure all eligible adults are invited for screening [2]. In the United Kingdom (UK), there are nationally organised screening programmes for breast, cervical and colorectal cancer, and these are estimated to save thousands of lives a year [3], [4], [5]. Despite their overall success, uptake of all three programmes is considered suboptimal [6], [7], [8]. In addition there are sociodemographic inequalities in attendance [8], [9], [10]. Improving access to screening and reducing inequalities are high on the cancer agenda [11].

A recent review of interventions in the context of organised programmes [12] found that cancer screening uptake could be increased by offering reminders, practitioner endorsement on the invitation or using alternative tests (e.g. human papillomavirus (HPV) testing). There was some evidence for using prescreening reminders, preset appointments, offering evening and weekend appointment times, mass media campaigns and direct contact with a health professional. However, in most cases room for improvement in attendance remains. An alternative approach to intervention design is to move away from using one-size-fits all interventions and consider how some interventions may be more effective for some groups than others, e.g. particular sociodemographic groups [13] or people with a certain screening history [14], [15]. While there are certainly interventions that may be effective at improving uptake for all groups, such as offering HPV self-testing for cervical cancer screening [16], [17], or face-to-face patient counselling for colorectal cancer screening [18], these may realistically be reserved for subgroups for which cheaper alternatives do not work.

**Fig. 1:**
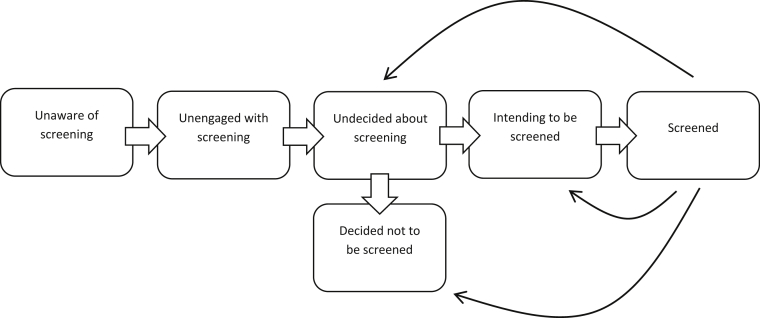
**An adapted version of the Precaution Adoption Process Model (PAPM) (Weinstein *et al.*, 2008)** demonstrating its use for explaining cancer screening behaviour in the context of an organised programme. Screening eligible men/women may be unaware of screening and once they become aware they may remain unengaged. After engaging with the screening decision they may remain undecided for an unspecified time before forming an intention to be screened or taking the decision not to be screened. People who intend to be screened may also remain at this stage for an unspecified time before actually participating. The need for repeated screening is indicated by the solid arrows which illustrate how those who have been screened may then become undecided, may decide not to participate next time, or may intend to participate in the next screening round.

Behavioural science can be used to better understand different types of decision-making for behaviours like participation or non-participation in cancer screening programmes. For example, an individual may never have been screened or may have been screened but not as recommended. Within both of these groups, motivations may also differ; individuals may be unaware they should be screened, be actively avoiding screening or be considering or preparing to be screened. One behavioural science model that lends itself to understanding screening non-participation is the Precaution Adoption Process Model (PAPM) [19]. The PAPM suggests people move through a series of stages towards participating in cancer screening (see Fig. 1). It highlights the role of past behaviour and differentiates between motives for non-attendance including informed decisions not to participate. It also acknowledges the importance of translating intention into action. This model has been used in the context of colorectal cancer screening in the United States of America (USA) [20], [21], [22].

The PAPM could be used to target appropriate interventions towards specific groups. Targeting interventions is more effective than using a single intervention for everyone without consideration of what a particular population needs. Using the PAPM to explore screening non-participation would help refine our understanding of screening non-participants, indicating which non-participant groups are the largest and where resources to improve participation are best placed. Identifying sociodemographic correlates of each non-participant type would indicate potential channels and content for targeted interventions. To our knowledge no one has used the PAPM to understand non-participation in an organised screening programme.

While this approach could be useful for all types of cancer screening, we have chosen to focus on cervical cancer screening non-participants. Breast screening coverage in England has improved over the last 10°years [7] and colorectal cancer screening is still relatively new in the UK, and is undergoing a number of changes. We therefore focussed on cervical cancer screening. The aims were: (1) to establish the percentage of British women classified into each cervical cancer screening non-participant type, as outlined by the PAPM and (2) to identify sociodemographic correlates with each non-participant type.

## 2 Methods

### 2.1 Participants

Data were collected by TNS (a market research agency) as part of their Omnibus survey, in which data are collected during one interview on behalf of multiple independent bodies. For each survey, TNS randomly select approximately 158 sampling points based on 2011 census data and the Postcode Address File. Interviewers approach households and invite eligible people to take part. At each location, preset quotas are set for gender, employment status and presence of children in the household. TNS do not provide a response rate.

We commissioned six waves of data collection estimating that this would achieve a sample of 3600 screening-eligible women (i.e. aged 25–64°years) across Great Britain. We anticipated that 13% (around 400 women) would be non-participants (based on previous surveys). This was expected to be sufficient for exploring sociodemographic differences between non-participant types. Data were collected in January/February 2016. Ethical approval was obtained from the University College, London (UCL) Research Ethics Committee (ref: 7585/001).

## 3 Measures

### 3.1 Screening history and future intention to be screened

**Fig. 2:**
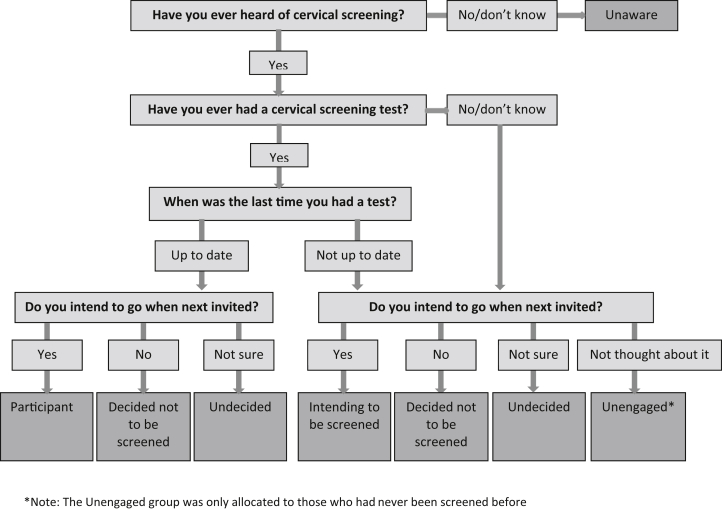
Algorithm allocating women to one of six stages in line with the PAPM.

Data were collected using face-to-face computer-assisted personal interviews (CAPIs). In Britain, women are invited for screening every 3°years (25- to 49-year-olds) or 5°years (50- to 64-year-olds). Four questions assessed past screening behaviour and future intention to be screened (see box 1), and an algorithm was used to classify women into one of six types of participation (see Fig. 2).

Box 1 Women were told ‘In the UK, women who are aged between 25 and 64 are invited to participate in the National Health Service (NHS) cervical screening program’ followed by ‘Have you ever heard of cervical screening, also known as the smear test or Pap test?’ (yes/no/don't know) and this was accompanied with a photograph of a woman being screened. Those who responded yes were also asked ‘Have you ever had a cervical screening test?’ (yes/know/don't know), and if applicable ‘When was the last time you had a cervical screening test?’ (within the last 3°years/3–5°years ago/longer than 5°years ago/don't know). All women who had heard of screening were also asked ‘Do you intend to go when next invited?’ (yes/no/don't know) with ‘I've never thought about it’ as an additional response for those who had not been screened before.

Women who were up-to-date and intending to be screened when next invited were classified as participants. The remaining women were classified as non-participants and fell into one of five types: Unaware, Unengaged, Undecided, Decided not to be screened, Intending to be screened. Most of these women were overdue for screening, but a few were up-to-date and were classified as non-participants because they had not formed strong intentions to attend in future (*n* = 42).

### 3.2 Sociodemographic characteristics

Sociodemographic variables were collected using items designed by TNS or based on the 2011 census. These included age, marital status, number of and age of children and social grade. Social grade represented the occupation of the Chief Income Earner in the household: AB managerial/professional; C1 supervisory; C2 skilled manual; D semi-skilled/unskilled manual; E casual/lowest grade workers [23]. We also assessed ethnicity (White British or Irish, White Other, South Asian, Black, Mixed or other ethnic background), and first language spoken (English or other).

## 4 Analysis

Data were analysed in SPSS v.22, version 22 of the statistical software package, originally known as 'Statistical Package for the Social Sciences (SPSS), now known as IBM SPSS Statistics (used for data collection, data mining, text analytics, batch and automated scoring services). TNS provide sampling weights to ensure that the data are population-representative in relation to age, social grade and region. All analyses were weighted using the complex samples function in SPSS. Binary logistic regression was used to determine sociodemographic differences between screening participants and non-participants. Multinomial logistic regression was then used to explore the odds of being each individual non-participant type (relative to the ‘Participants’) by sociodemographic group (unadjusted and adjusted). We have focussed on the results of the unadjusted analyses because understanding the sociodemographic characteristics of women in each non-participant type is useful for targeting interventions, regardless of whether associations are confounded by other variables.

## 5 Results

Data were collected from 3661 women. Those who reported having had a hysterectomy or cervical cancer (*n* = 369) and those over 60°years of age who lived in Scotland (*n* = 27) were excluded (cervical screening stops at 60°years in Scotland). We also excluded those who provided insufficient data to determine their screening stage (*n* = 152). Analyses were conducted with 3113 women (weighted *n* = 3111) aged 25–64 with a mean age of 43°years (*SE* = 0.20). See Table 1 for sample characteristics. Using the PAPM staging algorithm, 27% of women were classified as screening non-participants.

**Table 1:** Sample characteristics and proportion of each demographic group classified as screening non-participants.

	All (n = 3111)		Proportion of screening non-participants (n = 793)		OR (95% CI) for being a non-participant versus a participant
	n	%	n	%	
**Age**					
25–34	879	28	274	31	1.00
35–44	825	27	218	27	**0.79 (0.65–0.97)**
45–54	814	26	180	22	**0.62 (0.50–0.78)**
55–64	593	19	121	20	**0.57 (0.44–0.72)**
**Social grade**					
AB	860	28	155	18	1.00
C1	894	29	220	25	**1.49 (1.15–1.92)**
C2	642	21	155	24	**1.45 (1.10–1.90)**
D	440	14	151	34	**2.38 (1.82–3.13)**
E	275	8.9	112	41	**3.13 (2.34–4.17)**
**Working status**					
Working full-time	1233	40	280	23	1.00
Working part-time	909	29	207	23	1.00 (0.8.–1.24)
Not working	969	31	306	32	**1.57 (1.30–1.89)**
**Marital status**					
Currently married	2134	69	499	23	1.00
Previously married	370	11.9	97	23	1.17 (0.90–1.51)
Single	608	20	197	32	**1.57 (1.29–1.91)**
**Children under 5 years**					
No	2446	79	600	25	1.00
Yes	665	21	193	29	**1.26 (1.04–1.52)**
**Ethnicity**					
White British/Irish	2281	74	487	21	1.00
Any other white	354	11.4	96	27	**1.37 (1.06–1.77)**
South Asian	230	7.4	107	46	**3.18 (2.42–4.18)**
Black	148	4.8	65	44	**2.88 (2.04–4.07)**
Mixed/other ethnicity	89	2.9	33	37	**2.15 (1.35–3.41)**
**First language**					
English	2563	83	555	22	1.00
Other	523	17	214	41	**2.51 (2.07–3.06)**

OR = Odds Ratio, CI = Confidence interval.

ORs/CIs in bold indicate significance at p < .001.

### 5.1 Stages of non-participation

Among the 793 women who were classified as non-participants, most were unaware of screening (28%), intending to be screened but currently overdue (51%) or had decided not to be screened (15%). The proportions who were unengaged or undecided were much smaller (5% and 2% respectively; see Table 2). The following analyses explore the sociodemographic characteristics of each non-participant type with screening participants as the reference category (see Table 3, Table 4). Unadjusted analyses are described below. We excluded women who were undecided about screening as the numbers were small (*n* = 15).

**Table 2:** Sociodemographic characteristics of the five non-participant types (n = 793).

	Unaware	Unengaged	Undecided	Decided not to be screened	Intending to be screened
**All (row %)**	219 (28)	35 (4.5)	15 (1.9)	118 (15)	406 (51)
**Age**					
25–34	98 (45)	18 (51)	1 (20)	17 (15)	137 (34)
35–44	58 (27)	6 (17)	0 (0)	25 (21)	129 (32)
45–54	25 (11.3)	1 (4)	3 (23)	6 (5.3)	78 (19)
50–64	38 (17)	10 (28)	9 (57)	69 (59)	61 (15)
**Social grade**					
AB	32 (15)	5 (14)	3 (20)	24 (21)	91 (22)
C1	51 (25)	8 (23)	3 (19)	39 (33)	116 (29)
C2	38 (17)	9 (25)	1 (6.0)	18 (15)	89 (22)
D	57 (26)	5 (15)	4 (28)	19 (16)	66 (16)
E	38 (17)	8 (22)	4 (26)	17 (15)	45 (11)
**Working status**					
Working full-time	68 (31)	12 (34)	2 (15)	48 (41)	150 (37)
Working part-time	44 (20)	9 (25)	5 (35)	26 (22)	122 (30)
Not working	107 (49)	15 (41)	7 (50)	44 (38)	133 (33)
**Marital status**					
Currently married	142 (65)	21 (59)	9 (60)	52 (45)	274 (68)
Previously married	22 (10.2)	5 (14)	1 (5.5)	29 (25)	40 (9.8)
Single	55 (25)	10 (27)	5 (34)	36 (31)	91 (23)
**Children under 5 years**					
No	158 (72)	24 (67)	12 (81)	107 (90)	299 (74)
Yes	61 (28)	12 (33)	3 (19)	11 (9.6)	106 (26)
**Ethnicity**					
White British/Irish	80 (37)	17 (49)	11 (71)	90 (78)	289 (71)
Any other white	41 (19)	5 (13)	2 (10.6)	10 (8.8)	39 (9.6)
South Asian	53 (24)	6 (16)	1 (5.8)	8 (6.7)	40 (9.8)
Black	32 (15)	4 (9.9)	1 (4.8)	6 (5.5)	22 (5.5)
Mixed/other	11 (5)	4 (11.9)	1 (7.4)	2 (1.4)	15 (3.7)
**First language**					
English	92 (46)	23 (66)	11 (71)	98 (84)	331 (83)
Other	109 (54)	12 (34)	4 (29)	19 (17)	70 (17)

Note: column n (column percentage), except where specified.

**Table 3:** Odds of being in each non-participant group compared with the screening participant group (unadjusted multinomial logistic regression models).

	Unaware (n = 219)	Unengaged (n = 35)	Decided not to be screened (n = 118)	Intending to be screened (n = 406)
	OR (95% CI)	OR (95% CI)	OR (95% CI)	OR (95% CI)
**Age**				
25–34	1.00	1.00	1.00	1.00
35–44	**0.59 (0.43–0.81)**	**0.33 (0.13–0.81)**	1.47 (0.80–2.69)	0.94 (0.72–1.22)
45–55	**0.34 (0.23–0.52)**	0.41 (0.15–1.10)	1.35 (0.72–2.54)	**0.74 (0.56–0.99)**
55–64	**0.35 (0.23–0.54)**	**0.25 (0.08–0.74)**	**3.86 (2.24–6.66)**	**0.30 (0.20–0.45)**
**Social grade**				
AB	1.00	1.00	1.00	1.00
C1	**1.75 (1.07–2.87)**	1.75 (0.44–6.99)	1.69 (0.94–3.05)	1.34 (0.96–1.85)
C2	**1.72 (1.02–2.89)**	2.63 (0.68–10.14)	1.09 (0.55–2.14)	**1.42 (1.00–2.00)**
D	**4.31 (2.67–6.96)**	2.65 (0.66–10.56)	**1.93 (1.02–3.67)**	**1.77 (1.24–2.53)**
E	**5.11 (3.08–8.46)**	**6.83 (1.80–25.90)**	**3.07 (1.62–5.81)**	**2.15 (1.47–3.13)**
**Working status**				
Working full-time	1.00			
Working part-time	0.88 (0.59–1.32)	1.00 (0.38–2.65)	0.73 (0.43–1.24)	1.10 (0.84–1.45)
Not working	**2.25 (1.65–3.09)**	1.76 (0.82–3.76)	1.33 (0.86–2.05)	1.27 (0.99–1.63)
**Marital status**				
Single	**1.53 (1.12–2.10)**	1.81 (0.83–3.92)	**2.76 (1.75–4.34)**	**1.33 (1.03–1.72)**
Currently married	1.00	1.00	1.00	1.00
Previously married	0.95 (0.60–1.50)	1.44 (0.53–3.90)	**3.34 (2.06–5.43)**	0.87 (0.60–1.25)
**Children under 5 years**				
No	1.00	1.00	1.00	1.00
Yes	**1.51 (1.12–2.04)**	**1.96 (1.01–3.83)**	**0.41 (0.23–0.75)**	**1.39 (1.09–1.77)**
**Ethnicity**				
White British/Irish	1.00	1.00	1.00	1.00
Any other white	**3.51 (2.39–5.16)**	1.84 (0.71–4.82)	0.79 (0.41–1.49)	0.94 (0.65–1.36)
South Asian	**9.55 (6.52–14.00)**	**4.68 (1.89–11.56)**	1.26 (0.59–2.69)	**1.99 (1.37–2.89)**
Black	**8.63 (5.48–13.58)**	**4.34 (1.25–15.06)**	1.53 (0.64–3.65)	**1.67 (1.02–2.75)**
Mixed/other	**4.26 (2.13–8.50)**	**7.74 (2.11–28.38)**	0.56 (0.12–2.54)	1.67 (0.92–3.03)
**First language**				
English	1.00	1.00	1.00	1.00
Other	**7.73 (5.76–10.37)**	**3.36 (1.65–6.83)**	1.29 (0.78–2.13)	**1.37 (1.03–1.82)**

OR = Odds Ratio, CI = Confidence interval.

ORs/CIs in bold indicate significance at p < .001.

**Table 4:** Odds of being in each non-participant group compared with the screening participant group (fully adjusted model).

	Unaware OR (95% CI)	Unengaged OR (95% CI)	Decided not to be screened OR (95% CI)	Intending to be screened OR (95% CI)
**Age**				
25–34	1.00	1.00	1.00	1.00
35–49	**0.66 (0.48–0.91)**	0.30 (0.13–0.72)	1.33 (0.73–2.42)	1.08 (0.85–1.36)
50–64	**0.46 (0.31–0.68)**	0.52 (0.19–1.38)	**3.45 (2.00–5.95)**	**0.36 (0.25–0.50)**
**Ethnicity**				
White British/Irish	1.00	1.00	1.00	1.00
Any other white	**2.97 (2.03–4.36)**	1.73 (0.63–4.77)	1.20 (0.65–2.19)	0.80 (0.55–1.17)
South Asian	**8.12 (5.33–12.37)**	**4.37 (1.61–11.85)**	**2.31 (1.06–5.03)**	**1.64 (1.11–2.40)**
Black	**7.29 (4.60–11.56)**	**4.01 (1.14–14.11)**	1.36 (0.54–3.44)	1.34 (0.79–2.27)
Mixed/other	**4.31 (2.15–8.67)**	**8.11 (2.07–31.74)**	0.68 (0.15–3.20)	1.46 (0.79–2.70)
**Social grade**				
AB	1.00	1.00	1.00	1.00
C1/C2	1.43 (0.89–2.29)	1.78 (0.50–6.31)	1.26 (0.74–2.14)	1.31 (0.97–1.77)
DE	**3.81 (2.38–6.10)**	3.42 (0.94–12.43)	1.60 (0.92–2.78)	**1.84 (1.32–2.58)**
**Marital status**				
Single	1.07 (0.75–1.52)	1.23 (0.54–2.81)	**2.79 (1.70–4.59)**	1.12 (0.85–1.49)
Currently married	1.00	1.00	1.00	1.00
Previously married	1.35 (0.83–2.21)	1.75 (0.60–5.09)	**2.46 (1.51–4.00)**	1.13 (0.77–1.67)

OR = Odds Ratio, CI = Confidence interval.

ORs/CIs in bold indicate significance at p < .001.

Note: We excluded working status, children under 5 and language to avoid having an inadequate number of expected frequencies (i.e. because nearly all women with a child under 5 were <45 years; nearly all white British/Irish women spoke English as their first language; and almost all of the social grade E women were not working, by definition Social grade E includes the unemployed).

### 5.2 Age

Age was significantly associated with all four types of non-participation. Women in each of the age groups over 34°years were significantly less likely to be unaware of screening than women in the 25- to 34-year age group. A similar pattern was seen for being unengaged with screening. Older women were also less likely to be intending to be screened than those in the youngest group. Conversely, women aged 55–64°years were significantly more likely to have decided not to be screened than women in the youngest group.

### 5.3 Social grade and working status

Social grade was associated with all four types of non-participation. Compared with women in the highest social grade (AB), women from each of the lower social grades were more likely to be unaware and to be intending to be screened, with each reduction in social grade being associated with a corresponding increase in likelihood of being unaware or intending to be screened. Similar patterns appeared for the unengaged group and for those who had decided not to be screened. Compared with women who worked full-time, those who were not working were more likely to be unaware of cervical cancer screening.

### 5.4 Family structure (marital status, children)

Single women were more likely to be unaware of screening, to have decided not to be screened, or to be intending to be screened than married women. Previously married women were more likely to have decided not to attend. Compared with those without a child under the age of 5°years, women who had a child under 5 were more likely to be unaware of screening, unengaged with screening and to be intending to be screened, but less likely to have decided not to attend.

### 5.5 Ethnicity and first language

Compared with white British women, women from each ethnic minority group were more likely to be unaware of screening. A similar pattern was seen for the unengaged group. In addition, South Asian and Black women were more likely to be intending to be screened than white British women. Women with English as a second language were more likely to be unaware, unengaged and to be intending to be screened. After adjusting for language, there were no longer any differences between white British women and women from other white or mixed ethnic backgrounds.

## 6 Discussion

This study advances our understanding of cancer screening non-participation by classifying non-participants into different types. Using the PAPM as a framework, we identified three main types of non-participants, and we suggest that these types should be the focus of interventions to improve informed uptake.

The largest group of non-participants were those who intended to go for screening but were currently overdue. Previous studies have shown that intending to go but not getting around to it is one of the most commonly endorsed reasons for not attending among overdue women [24], and the gap between intention and behaviour has long been recognised in behavioural science and has previously been demonstrated for cervical screening [25]. However, this is the first study to suggest that this accounts for such a large proportion on non-attendance (around half). Women who were 25–35 years old, those who were single and those from lower social grades were disproportionately likely to be in this group. Interventions designed to encourage action among these women may involve ‘nudge’ style techniques [26] such as additional reminders, which have already been suggested to improve uptake among young women [17]. Small-scale studies have shown that asking women to plan when, where and how they would make an appointment (referred to as forming an implementation intention) was effective for women who intended to go for screening [27]. Changing the screening infrastructure could also nudge women who intend to be screened, for example in a recent UK-based randomised controlled trial (RCT) timed appointments improved uptake among non-attenders for their first invitation [28].

The second largest group of non-participants was unaware of screening. It is surprising that such a large proportion of women have not heard of cervical cancer screening when everyone should have received a screening invitation accompanied by a leaflet as part of the NHS programme. The PAPM argues that awareness is the first stage necessary before behaviour can occur, and awareness is, of course, essential to informed choice. Work in the context of colorectal cancer screening suggests that many of those who have never been screened have not read any of the information sent to them [29]. Unaware women were more likely to be younger and from lower social grade and/or ethnic minority backgrounds, consistent with previous studies [30], [31], [32]. Interventions aimed at raising awareness of cervical cancer screening are likely to be beneficial for a significant proportion of women, yet written materials may not be sufficient for this (as they are already used). Awareness campaigns using other channels, such as TV, radio and social media, or community outreach, may be a more effective approach for this group. An early RCT showed that in a diverse community, face-to-face visits with an outreach worker were more effective at increasing cervical screening uptake than written materials alone [33].

The third biggest group of non-participants in our survey was those who had decided not to be screened. These women tended to be older and many had been screened before. Further exploration of why this group has decided not to be screened will help inform the content of interventions for these women. Deciding not to be screened is, of course, a legitimate decision providing it is made on the basis of informed choice. If women have decided not to attend because of a dislike for the test, offering alternative tests such as an HPV self-test or non-speculum testing, may overcome these concerns [35], [36].

### 6.1 Limitations

While the survey sample was broadly population representative and the cervical screening rates in the study were similar to those reported by the national screening programme [6], TNS do not collect information needed to calculate a response rate or collect any data from non-responders. Because the survey was carried out within an omnibus, and therefore was not described to participants as being health- or screening-related, systematic bias due to interest in or beliefs about this topic is unlikely. Moreover, interviews are carried out in the evening as well as the daytime, and there are quotas to ensure non-working women are not over-represented. However, it is likely that some participation biases remain. We relied on self-reported screening uptake, and while this is how screening is predominantly measured in surveys, social desirability bias may lead to underestimates of non-participation or time since last screening test [37]. A small group of women were aware of screening but had not engaged with the decision to attend. While we included these in analyses as a separate group, the lack of significant difference for this group could be due to small numbers. While the PAPM includes longitudinal aspects, the data presented here were collected cross-sectionally. Further research using a longitudinal design would add support to the use of the PAPM as a means of classifying non-participants.

## 7 Conclusion

This work suggests that the vast majority of women in Britain who are not participating in cervical screening as recommended are not making an active decision not to attend. Most non-participants are either unaware or would like to be screened but are unable to translate their positive intentions to be screened into action. Drawing together the current findings with those in the USA we suggest the PAPM is a useful way to distinguish between non-participant types. By identifying demographic differences between non-participant types, we provide important information for screening providers about how they might tackle low uptake. Further exploration of attitudinal differences across different non-participant types may provide useful guidance on the content of these targeted interventions.

## Role of the funding source

The study sponsor had no role in the study design, conduct or interpretation of the data or the writing of the report and decision to submit for publication.

## Conflict of interest statement

None declared.

## References

[1] Wilson J., Jungner G. (1968). Principles and practice of screening for disease.

[2] Miles A., Cockburn J., Smith R.A., Wardle J. (2004). A perspective from countries using organized screening programs. Cancer.

[3] Landy R., Pesola F., Castanon A., Sasieni P. (2016). Impact of cervical screening on cervical cancer mortality: estimation using stage-specific results from a nested case-control study. Br J Cancer.

[4] Duffy S.W., Tabar L., Olsen A.H., Vitak B., Allgood P.C., Chen T.H. (2010). Absolute numbers of lives saved and overdiagnosis in breast cancer screening, from a randomized trial and from the Breast Screening Programme in England. J Med Screen.

[5] Hewitson P., Glasziou P., Watson E., Towler B., Irwig L. (2008). Cochrane systematic review of colorectal cancer screening using the fecal occult blood test (hemoccult): an update. Am J Gastroenterol.

[6] Screening and Immunisations Team, Health and Social Care Information Centre. Cervical Screening Programme, England: Statistics for 2014–15. http://content.digital.nhs.uk/catalogue/PUB18932/nhs-cervical-stat-eng-2014-15-rep.pdf [Accessed 12 December 2016].

[7] Screening and Immunisations Team, Health and Social Care Information Centre. Breast Screening Programme, England: Statistics for 2014–15. http://content.digital.nhs.uk/catalogue/PUB20018/bres-scre-prog-eng-2014-15-rep.pdf [Accessed 12 December 2016].

[8] von Wagner C., Baio G., Raine R., Snowball J., Morris S., Atkin W. (2011). Inequalities in participation in an organized national colorectal cancer screening programme: results from the first 2.6 million invitations in England. Int J Epidemiol.

[9] Moser K., Patnick J., Beral V. (2009). Inequalities in reported use of breast and cervical screening in Great Britain: analysis of cross sectional survey data. BMJ.

[10] Douglas E., Waller J., Duffy S.W., Wardle J. (2016). Socioeconomic inequalities in breast and cervical screening coverage in England: are we closing the gap?. J Med Screen.

[11] DH (2011). Improving outcomes: a strategy for cancer. https://www.gov.uk/government/uploads/system/uploads/attachment_data/file/213785/dh_123394.pdf.

[12] Duffy S.W., Myles J.P., Maroni R., Mohammad A. (2016). Rapid review of evaluation of interventions to improve participation in cancer screening services. J Med Screen.

[13] Wardle J., von Wagner C., Kralj-Hans I., Halloran S.P., Smith S.G., McGregor L.M. (2016). Effects of evidence-based strategies to reduce the socioeconomic gradient of uptake in the English NHS Bowel Cancer Screening Programme (ASCEND): four cluster-randomised controlled trials. Lancet.

[14] Stein K., Lewendon G., Jenkins R., Davis C. (2005). Improving uptake of cervical cancer screening in women with prolonged history of non-attendance for screening: a randomized trial of enhanced invitation methods. J Med Screen.

[15] White B., Power E., Ciurej M., Lo S.H., Nash K., Ormiston-Smith N. (2015). Piloting the impact of three interventions on Guaiac faecal occult blood test uptake within the NHS bowel cancer screening programme. Biomed Res Int.

[16] Sultana F., English D.R., Simpson J.A., Drennan K.T., Mullins R., Brotherton J.M. (2016). Home-based HPV self-sampling improves participation by never-screened and under-screened women: results from a large randomized trial (iPap) in Australia. Int J Cancer.

[17] Albrow R., Blomberg K., Kitchener H., Brabin L., Patnick J., Tishelman C. (2014). Interventions to improve cervical cancer screening uptake amongst young women: a systematic review. Acta Oncol.

[18] Baker D.W., Brown T., Buchanan D.R., Weil J., Balsley K., Ranalli L. (2014). Comparative effectiveness of a multifaceted intervention to improve adherence to annual colorectal cancer screening in community health centers: a randomized clinical trial. JAMA Intern Med.

[19] Weinstein N., Sandman P., Blalock S., Glanz K., Rimer B., Viswanath K. (2008). The precaution adoption process model. Health behavior and health education.

[20] Costanza M.E., Luckmann R., Stoddard A.M., Avrunin J.S., White M.J., Stark J.R. (2005). Applying a stage model of behavior change to colon cancer screening. Prev Med.

[21] Ferrer R.A., Hall K.L., Portnoy D.B., Ling B.S., Han P.K., Klein W.M. (2011). Relationships among health perceptions vary depending on stage of readiness for colorectal cancer screening. Health Psychol.

[22] Hester C.M., Born W.K., Yeh H.W., Young K.L., James A.S., Daley C.M. (2015). Decisional stage distribution for colorectal cancer screening among diverse, low-income study participants. Health Educ Res.

[23] Ipsos (2009). Social grade: a classification tool. https://www.ipsos-mori.com/DownloadPublication/1285_MediaCT_thoughtpiece_Social_Grade_July09_V3_WEB.pdf.

[24] Waller J., Bartoszek M., Marlow L., Wardle J. (2009). Barriers to cervical cancer screening attendance in England: a population-based survey. J Med Screen.

[25] Sheeran P., Hewstone M., Stroebe W. (2002). Intention-behaviour relations: a conceptual and empirical review. European review of social psychology.

[26] Thaler R.H., Sunstein C.R. (2008). Nudge: improving decisions about health.

[27] Sheeran P., Orbell S. (2000). Using implementation intentions to increase attendance for cervical cancer screening. Health Psychol.

[28] Kitchener H.C., Gittins M., Rivero-Arias O., Tsiachristas A., Cruickshank M., Gray A. (2016). A cluster randomised trial of strategies to increase cervical screening uptake at first invitation (STRATEGIC). Health Technol Assess.

[29] Kobayashi L.C., Waller J., von W.C., Wardle J. (2016). A lack of information engagement among colorectal cancer screening non-attenders: cross-sectional survey. BMC Public Health.

[30] Low E.L., Simon A.E., Lyons J., Romney-Alexander D., Waller J. (2012). What do British women know about cervical cancer symptoms and risk factors?. Eur J Cancer.

[31] Robb K., Wardle J., Stubbings S., Ramirez A., Austoker J., Macleod U. (2010). Ethnic disparities in knowledge of cancer screening programmes in the UK. J Med Screen.

[32] Marlow L.A., Wardle J., Waller J. (2015). Understanding cervical screening non-attendance among ethnic minority women in England. Br J Cancer.

[33] McAvoy B.R., Raza R. (1991). Can health education increase uptake of cervical smear testing among Asian women?. BMJ.

[35] Verdoodt F., Jentschke M., Hillemanns P., Racey C.S., Snijders P.J., Arbyn M. (2015). Reaching women who do not participate in the regular cervical cancer screening programme by offering self-sampling kits: a systematic review and meta-analysis of randomised trials. Eur J Cancer.

[36] Fargnoli V., Petignat P., Burton-Jeangros C. (2015). To what extent will women accept HPV self-sampling for cervical cancer screening? A qualitative study conducted in Switzerland. Int J Womens Health.

[37] Klungsoyr O., Nygard M., Skare G., Eriksen T., Nygard J.F. (2009). Validity of self-reported Pap smear history in Norwegian women. J Med Screen.

